# Comparison of the COM-FCP inclination angle and other mediolateral stability indicators for turning

**DOI:** 10.1186/s12938-017-0325-z

**Published:** 2017-03-24

**Authors:** Rui Xu, Xin Wang, Jiajia Yang, Feng He, Xin Zhao, Hongzhi Qi, Peng Zhou, Dong Ming

**Affiliations:** 1 Department of Biomedical Engineering, College of Precision Instruments and Optoelectronics Engineering, Tianjin University, Tianjin, China; 2 Tianjin Key Laboratory of Biomedical Detecting Techniques and Instruments, Tianjin University, Tianjin, China

**Keywords:** Circular walking, Curvature, Mediolateral stability, Mediolateral inclination angle, Step length

## Abstract

**Background:**

Studies have shown that turning is associated with more instability than straight walking and instability increases with turning angles. However, the precise relationship of changes in stability with the curvature and step length of turning is not clear. The traditional center of mass (COM)-center of pressure (COP) inclination angle requires the use of force plates. A COM-foot contact point (FCP) inclination angle derived from kinematic data is proposed in this study as a measure of the stability of turning.

**Methods:**

In order to generate different degrees of stability, we designed an experiment of walking with different curvatures and step lengths. Simultaneously, a novel method was proposed to calculate the COM-FCP inclination angles of different walking trajectories with different step lengths for 10 healthy subjects. The COM-FCP inclination angle, the COM acceleration, the step width and the COM-ankle inclination angles were statistically analyzed.

**Results:**

The statistical results showed that the mediolateral (ML) COM-FCP inclination angles increased significantly as the curvature of the walking trajectories or the step length in circular walking increased. Changes in the ML COM acceleration, the step width and the ML COM-ankle inclination angle verified the feasibility and reliability of the proposed method. Additionally, the ML COM-FCP inclination angle was more sensitive to the ML stability than the ML COM-ankle inclination angle.

**Conclusions:**

The work suggests that it is more difficult to keep balance when walking in a circular trajectory with a larger curvature or in a larger step length. Essentially, turning with a larger angle in one step leads to a lower ML stability. A novel COM-FCP inclination angle was validated to indicate ML stability. This method can be applied to complicated walking tasks, where the force plate is not applicable, and it accounts for the variability of the base of support (BOS) compared to the COM-ankle inclination angle.

## Background

Turning is a necessary aspect of functional ambulation, allowing avoidance of obstacles and navigation of corners. Up to 50% of all walking steps involve turning [1]. Kinematic analysis indicates that turning is less stable than straight walking as it demands constant body reorientation [2–4]. The majority of the mobility-impaired population has difficulty in turning, especially patients with Parkinson’s disease and stroke and older adults [5, 6]. In view of the risks of falling, the stability of turning warrants analysis and investigation. It has been reported that a larger turning angle is related to a higher risk of falling [7], and that the stability of turning decreases as the turning angle increases. However, little is known about how the curvature and step length of turning affect stability. Further understanding may facilitate the development of effective means of detecting those at risk of falls and the determination of the efficacy of medical interventions [8].

Gait stability, defined as the ability to maintain gait in perturbations, has been quantified using different methods, such as the maximum Lyapunov exponents [9], step length and width [10, 11] and the COM mediolateral (ML) movement [12]. The ML acceleration of COM has been used to detect the falling risks [13] and imbalance during walking [4, 14]. It is a clinically important measurement to potentially indicate the ability to safely ambulate at higher risk of falling [4].

Traditionally, the projection of the COM has to be confined within the base of support (BOS) to maintain balance [15]. In this condition, the movement of COM relative to the center of pressure (COP) has been widely used for the studies of the body’s stability during activities [15–18] as the COP can be obtained from a force plate directly and accurately. The COM-COP inclination angles were studied and quantified in the work of Lee and Chou [19]. It was found that the COM-COP inclination angle identified elderly people with imbalance and the frontal plane inclination angle was independent of walking speed and activity level.

However, the use of the force plate to obtain COP leads to a constrained walking trajectory and inconvenience for those with motor disorders as they usually have smaller step lengths [20]. Additionally, informing the subjects to target the force plate and achieve a clean foot placement may alter natural gait performance [21]. To overcome these problems, the lateral ankle marker was used to obtain the COM-ankle inclination angle [21]. However, this measure is not available for double stance phase and does not describe the variability of the BOS during the whole gait cycle. Therefore, we have developed a new method to estimate the center of the BOS using foot contact points (FCP). The advantages of this proposed method are that it obviates the need for force plates and accounts for the variability of BOS during the whole gait cycle.

The objectives of this study were (1) to propose a method that can calculate the COM-FCP inclination angles based on kinematic data, (2) to validate the proposed method with experiments, in which gait stability varies with curvature and step length, and (3) to compare the proposed method with the COM acceleration, the step width and the COM-ankle inclination angle, among which the COM acceleration and the step width are believed to be reliable stability indicators.

## Methods

### Participants and experiment

Ten healthy subjects (7 females, 3 males; 22 ± 2 years old; no dyskinesia) were recruited from Tianjin University.

**Fig. 1:**
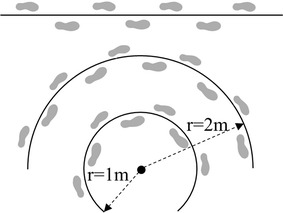
Walking trajectories. The three trajectories from *top* to *bottom* are for straight walking and circular walking (r = 2 m and r = 1 m), respectively

Before experiment, each subject was asked to walk with a self-chosen pace along a 5-m straight trajectory. The subject walked from the start to the end of the walkway. The walking distance of each subject *l* was measured from the first heel strike position to the last heel strike position just before the end of the walkway, in order to cover *n* complete steps. Here, *l* was less than 5 m and varied between subjects. The normal step length (NSL) of a subject was obtained as *l*/*n*. During the experiment, subjects walked along a straight trajectory (straight walking, SW) and two circular trajectories with radii of 2 m and 1 m (circular walking, CW2m or CW1m) with different step lengths: 60% (smaller step length, SSL), 100 and 120% (larger step length, LSL) of the NSL. The trajectories are shown in Fig. 1.

For each walking task except the NSL, the steps were indicated by footprints and the footprints were put on the two sides of the trajectory according to the required step length. These dual tasks required turning and following the footprints simultaneously. In order to weaken the distraction of the footprints, practice was performed to familiarize the subjects with the trials of SSL and LSL. Additionally, as the purpose of the study was not to quantify step length, it was not necessary for the subjects to adhere to the footprints strictly. These two steps were taken to avoid changing the walking direction and the step width of their natural gaits. For circular walking, every subject walked clockwise with the right foot inside the circle and the left outside. The number of steps for straight and circular walking depended on the step length applied. At least one gait cycle from left foot strike to the next left foot strike was covered.

### Data collection and processing

**Fig. 2:**
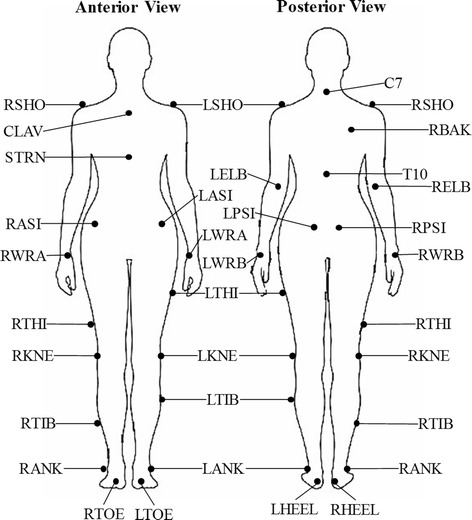
Placement of markers

During the experiment, the motion data of different walking tasks was recorded at 100 Hz using a 6-camera VICON motion analysis system (Vicon Motion Systems Ltd, Oxford, UK). As required, 29 markers, shown in Fig. 2, were used to locate the whole body.

The coordinates of the COM were output by Nexus (Vicon Motion Systems Ltd, Oxford, UK) with 29 markers’ coordinates. The positive direction of the z-axis was upward perpendicular to the floor. The positive directions of the x- and y-axes were roughly the anterior and the right of the subject during straight walking.

The coordinates were 4 Hz low-pass filtered using a fourth-order zero-phase Butterworth digital filter before analysis to remove noise and then segmented by gait cycles from left foot strike to the next left foot strike. Variables were extracted from the data of each gait cycle and averaged across all the complete gait cycles of one task.

### Inclination angles

The FCP (vertical axis *z* = 0) was calculated according to 4 markers on the feet, i.e. LHEEL, LTOE, RHEEL and RTOE. It was only used to indicate the general variability of the BOS. Its coordinates were obtained by (1).

1 $$C_{FCP} = \left( {\frac{{\sum\nolimits_{i} {C_{i} \cdot a_{i} } }}{{a_{LHEEL} + a_{LTOE} + a_{RHEEL} + a_{RTOE} }}} \right) \cdot I_{xy}$$

where

2 $$a_{i} = \left\{ {\begin{array}{*{20}c} 1& {\text{if the body part i is on the floor}}& \\ 0 & {\text{else}} \\ \end{array} } \right.$$

3 $$I_{xy} = \left[ {\begin{array}{*{20}c} 1 & 0 & 0 \\ 0 & 1 & 0 \\ 0 & 0 & 0 \\ \end{array} } \right]$$

and *C*_*i*_ represents the coordinates of marker *i* (*i* = LHEEL, LTOE, RHEEL or RTOE).

**Fig. 3:**
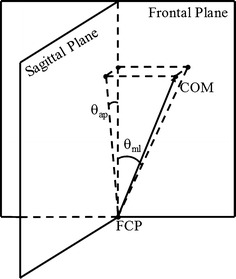
The inclination angles in the frontal and sagittal planes. The mediolateral (ML) inclination angle *θ*_*ml*_ is the projection of the angle between *FCP_COM* (the vector from *FCP* pointing to *COM*) and the vertical axis in the frontal plane and the anterioposterior (AP) inclination angle *θ*_*ap*_ is the projection of the same angle in the sagittal plane

The frontal plane was determined through the positions of two markers on the left and right shoulders (LSHO and RSHO) and perpendicular to the floor. The COM-FCP inclination angle is shown in Fig. 3.

### ML COM acceleration, step width and COM-ankle inclination angle

The COM acceleration was defined as the temporal rate of change in COM velocity. It was an effective parameter for the measure of balance ability, as it tracked body movements during gait [22]. The COM acceleration $a_{COM}$ was calculated as (4) and (5).

4 $$v_{COM} \left( t \right) = \frac{{C_{COM} \left( {t + 1} \right) - C_{COM} \left( t \right)}}{\Delta t}$$

5 $$a_{COM} \left( t \right) = \frac{{v_{COM} \left( {t + 1} \right) - v_{COM} \left( t \right)}}{\Delta t}$$

where *t* is the time frame, $\Delta t$ = 0.01 s, $C_{COM}$ is the coordinate of the COM, and $v_{COM}$ is the velocity of COM.

The ML COM acceleration $a_{COM\_ML}$ in this study was the projection of the COM acceleration $a_{COM}$ in the ML direction, which was determined as the horizontal component of the vector from LSHO to RSHO in (6).

6 $$a_{COM\_ML} = a_{COM} \cdot \frac{{\left( {C_{RSHO} - C_{LSHO} } \right) \cdot I_{xy} }}{{norm\left[ {\left( {C_{RSHO} - C_{LSHO} } \right) \cdot I_{xy} } \right]}}$$

where $C_{RSHO}$ and $C_{LSHO}$ are the coordinates of LSHO and RSHO.

**Fig. 4:**
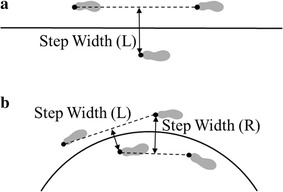
Definition of the step width. The step widths for straight walking (**a**) and circular walking (**b**)

The step width or stance width was defined as the shortest distance between a line connecting two successive foot prints of one limb and the foot print of the contralateral limb [23]. The step width (L) was calculated as the distance between the right heel marker at right foot strike and a line through the left heel markers at left foot strikes (see Fig. 4). However, as the circular walking was asymmetrical, the step width (R) in circular walking, which was calculated as the distance between the left heel marker at left foot strike and the line through the right heel markers at the successive right foot strikes, was larger than the step width (L). As the step width (L) and step width (R) varied inversely when the heading angle increased [23], the two step widths were both analyzed in this study.

These variables the ML COM acceleration, the step width and the ML COM-ankle inclination angle, were analyzed to verify the accuracy of the proposed method, but only the ML COM-ankle inclination angles in single stance phase were obtained.

### Statistical analysis

The variables mentioned above, including the maximal ML COM-FCP inclination angle [ML MAX (FCP)], the maximal ML COM acceleration (ML COM ACC MAX), the step width (L), the step width (R) and the maximal ML COM-ankle inclination angle [ML MAX (ankle)], were analyzed statistically.

All statistical analyses were performed using SPSS (version 22). The two-way (curvature × step length) repeated measures ANOVA was used to determine whether there was a significant effect caused by curvature, step length or the two factors together. A simple effect analysis was applied to assess the statistical differences between different curvatures or step lengths when there was a significant interaction between the two elements. The paired *t* test was used to assess the statistical differences when there were only significant main effects.

ML COM ACC MAX, step width (L), step width (R) and ML MAX (ankle) for different curvatures and step lengths were compared to determine any changes in walking stability. ML MAX (FCP) of different tasks were statistically compared to validate the use of ML MAX (FCP) in the determination of turning stability.

## Results

### COM acceleration and step width

**Fig. 5:**
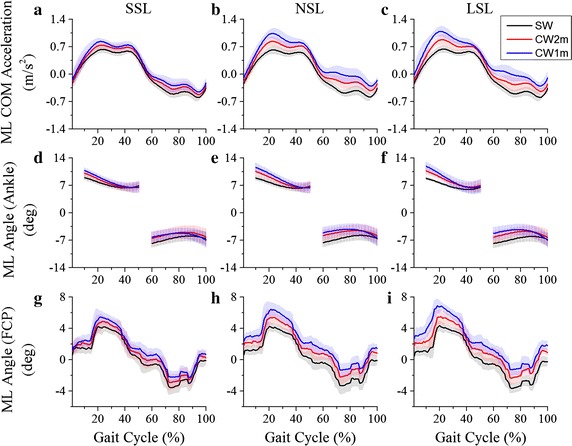
Curves of the ML COM acceleration, the ML angle (ankle) and the ML angle (FCP). The curves are the average values of ML COM acceleration for SSL (**a**), NSL (**b**) and LSL (**c**), the ML angle (ankle) for SSL (**d**), NSL (**e**) and LSL (**f**), and the ML angle (FCP) for SSL (**g**), NSL (**h**) and LSL (**i**) for different walking trajectories. *Shaded area* standard deviation

**Fig. 6:**
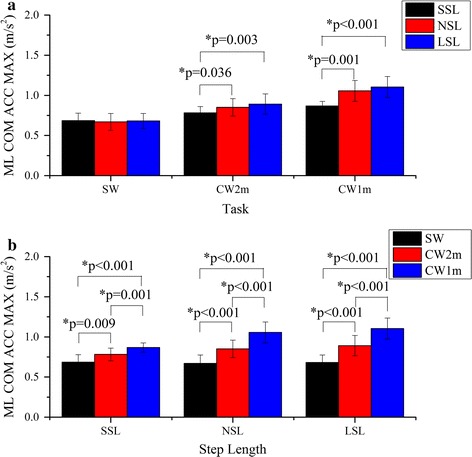
Comparison of the ML COM ACC. The ML COM ACC were compared by simple effect analysis between any two of the step lengths for certain walking trajectory (**a**) or between any two of the walking trajectories for certain step length (**b**). *Asterisk* statistical significance with p < 0.05

The mean curves of the ML COM acceleration of different tasks are shown in Fig. 5a–c. There was a significant interaction between the curvature and the step length on ML COM ACC MAX (F = 25.12, p < 0.001). The result of the simple effect analysis of ML COM ACC MAX is shown in Fig. 6. The ML COM ACC MAX of NSL and LSL were significantly greater than those of SSL for CW2m and CW1m (Fig. 6a); and the ML COM ACC MAX increased significantly with the curvature of the trajectory in a certain step length (Fig. 6b).

**Fig. 7:**
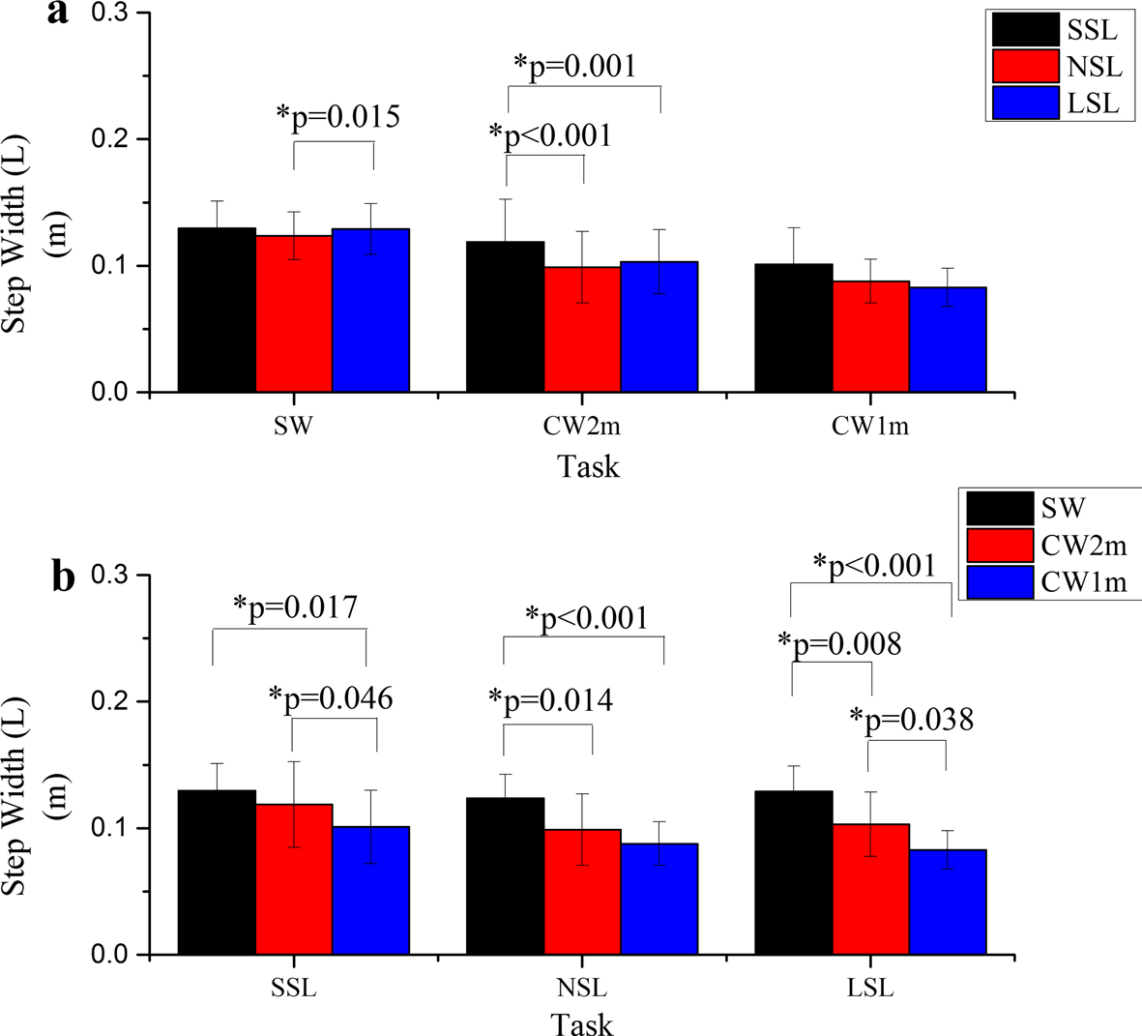
Comparison of the step width (L). The step width (L) were compared by paired t test between any two of the step lengths for certain walking trajectory (**a**) or between any two of the walking trajectories for certain step length (**b**). *Asterisk* statistical significance with p < 0.05

There were significant main effects of the curvature (F = 14.82, p = 0.003) and the step length (F = 10.33, p = 0.007) on step width (L). The result of the paired t test is shown in Fig. 7: step width (L) decreased significantly as curvature increased (Fig. 7b) and the step widths of SSL were significantly larger than those of NSL and LSL for CW2m (Fig. 7a).

**Fig. 8:**
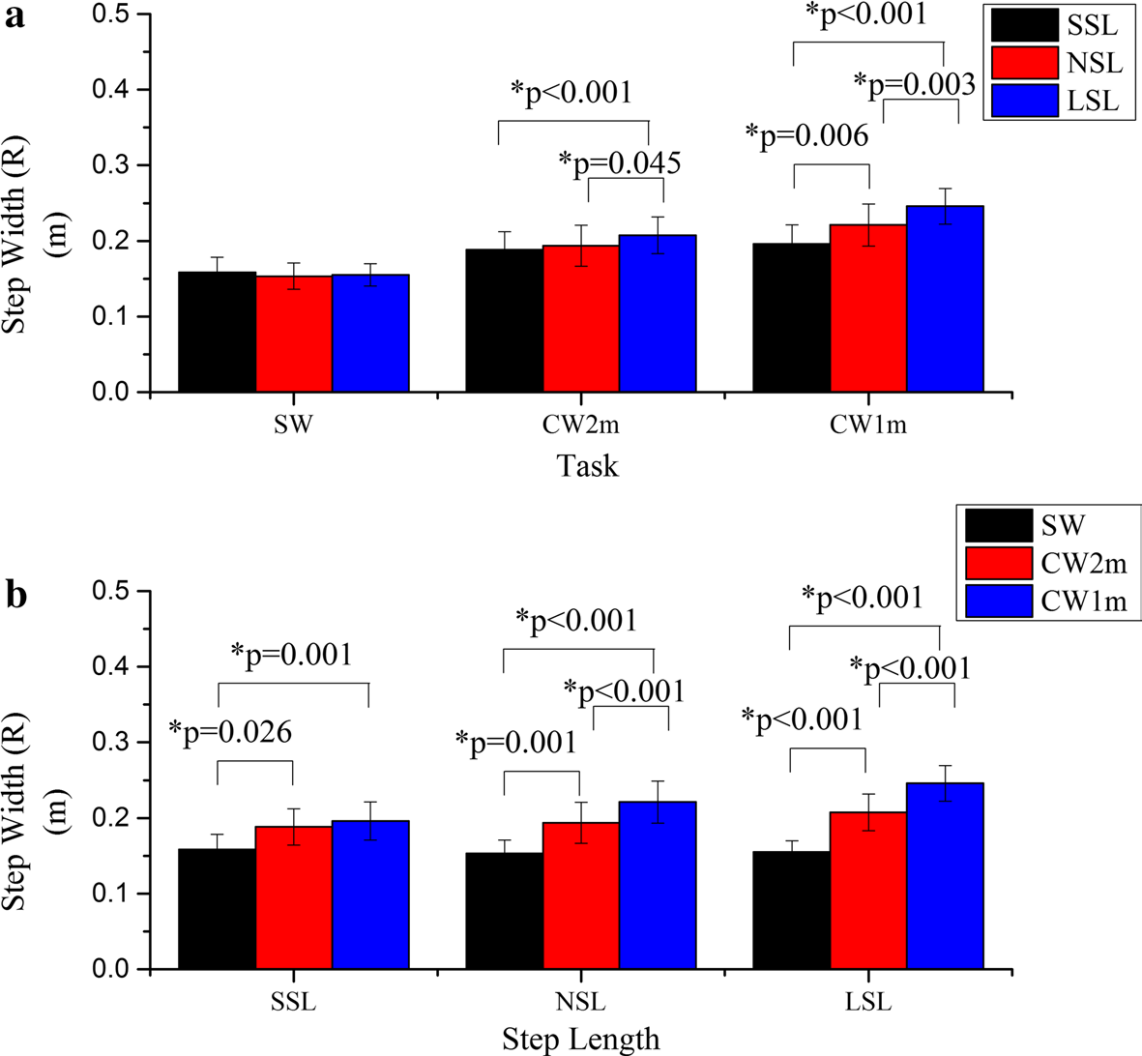
Comparison of the step width (R). The step width (R) were compared by simple effect analysis between any two of the step lengths for certain walking trajectory (**a**) or between any two of the walking trajectories for certain step length (**b**). *Asterisk* statistical significance with p < 0.05

There was a significant interaction between curvature and step length on step width (R) (F = 38.90, p < 0.001). The step width (R) of LSL was significantly larger than those of SSL and NSL for CW2m and it increased significantly with the step length for CW1m (Fig. 8a). Step width (R) increased significantly with curvature for certain step lengths but there was no significant difference between the step widths of CW2m and CW1m for SSL (Fig. 8b).

### Inclination angles

The mean ML angles of 10 subjects with the ankle marker and the FCP are shown in Fig. 5d–i. ML angles of these two methods decreased during the single stance phase of the left leg (the first single stance phase) and increased during the single-stance phase of the right leg (the second single stance phase).

The ML angles based on the ankle marker [ML Angle (ankle), Fig. 5d–f] had no definite peak values as the curves were separated by the double stance phase. Therefore, the ML MAX (ankle) was obtained by the mean value of the curve during the first single stance phase.

**Fig. 9:**
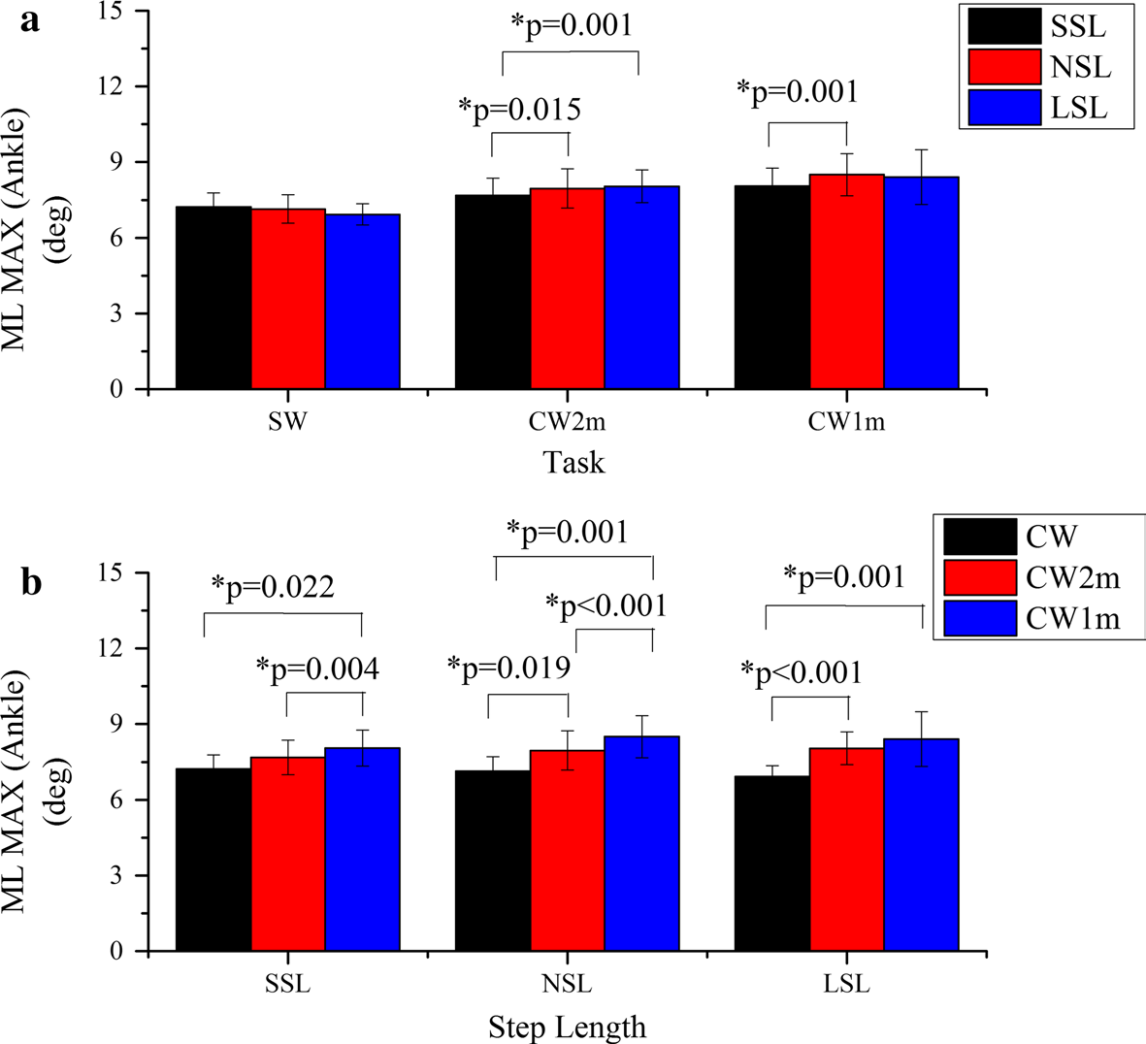
Comparison of the ML MAX (ankle). The ML MAX (ankle) were compared by simple effect analysis between any two of the step lengths for certain walking trajectory (**a**) or between any two of the walking trajectories for certain step length (**b**). *Asterisk* statistical significance with p < 0.05

**Fig. 10:**
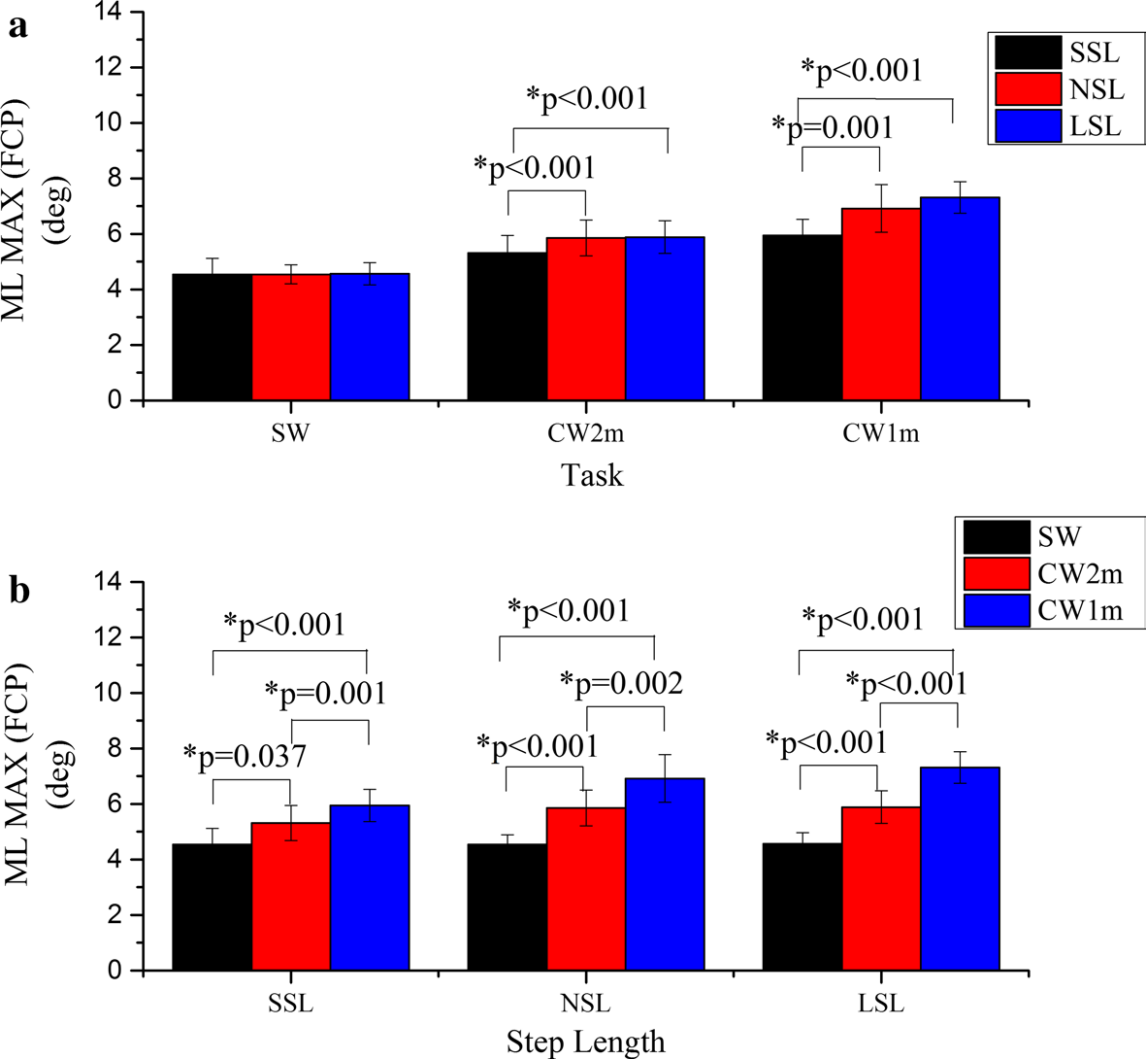
Comparison of the ML MAX (FCP). The ML MAX (FCP) were compared by simple effect analysis between any two of the step lengths for certain walking trajectory (**a**) or between any two of the walking trajectories for certain step length (**b**). *Asterisk* statistical significance with p < 0.05

There were significant interactions between the curvature and the step length on ML MAX (ankle) (F = 5.63, p = 0.001) and ML MAX (FCP) (F = 14.36, p < 0.001). The results of the simple effect analysis on ML MAX (ankle) and ML MAX (FCP) are shown in Figs. 9 and 10 respectively. The ML MAX (ankle) of SSL is significantly smaller than those of NSL for CW2m and CW1m and it was also significantly smaller than that of LSL for CW2m (Fig. 9a). The ML MAX (ankle) also increased significantly with curvature for different step lengths (Fig. 9b). As with the result of the ML MAX (ankle), the ML MAX (FCP) of SSL was significantly smaller than those of NSL and LSL for CW2m and CW1m (Fig. 10a) and the ML MAX (FCP) also increased significantly with curvature for different step lengths (Fig. 10b).

## Discussion

This study compared a novel method of computing the COM- FCP inclination angle with COM acceleration, step width and COM-ankle inclination angle. The specifically-designed experiment with varying walking step lengths and turning curvatures generated different degrees of walking stability for this comparison. The main findings of the present study can be summarized as: (1) stable turning can be achieved with shorter steps or smaller turning curvatures; (2) the proposed ML COM- FCP inclination angle is able to estimate the stability of turning and it is more sensitive than the ML COM-ankle inclination angle; (3) step width during the outer foot stance phase becomes larger as ML turning stability decreases.

Step length is an important gait parameter as it is often adjusted when crossing an obstacle. Usually, the change in step length alters anterioposterior stability, but has no significant effect on ML stability [24]. However, the situation is different during circular walking. A longer step in turning means a larger turning angle within one step, and so does a larger curvature. Evidence suggests that larger turning angles are related to a higher risk of falling [7]. Therefore, longer steps and sharper turns can cause a decrease in walking stability.

The COM acceleration is a commonly-used parameter to indicate stability [22, 25], while step width is a controversial stability indicator which is discussed below. The proposed COM- FCP inclination angle was developed based on the COM-ankle inclination angle, so the COM-ankle inclination angle was analyzed as a contrast.

COM acceleration and step width reflected the motion of COM and the area of BOS respectively. They have been reported to be indicative of the body’s balance alone [11, 25]. The COM-ankle inclination angle indicated the motion of COM relative to the ankle marker during single stance phase, which considered the COM and BOS together [21]. These three kinematic variables were related to COM and/or BOS and were validated as stability indicators. Therefore, these three variables were analyzed in this study.

It was found that increased ML COM acceleration predicted reduced balance [4]. From a biomechanical point of view, a larger ML COM acceleration means a larger centripetal force applied to the body, which is the driver of turning. It is expected that as with the turning angle increases (due to an increase in curvature or step length), the maximal absolute value of the ML COM acceleration increases significantly.

It is accepted that the ML COM acceleration of turning is larger than that of straight walking as turning requires extra acceleration to alter direction. However, the relationship between step width and walking stability is still unclear. Voluntary walking with a larger step width has been shown to increase the lateral stability [24]. Another study found a preferred step width decreased when applying lateral stabilization [26], suggesting that the choice of wide steps was a compensation for lateral instability. An increased step width may be predictive of falling [27] and associated with greater ML instability. Theoretically, a wider step indicates a larger area of BOS, but it is only likely true during double stance phase [27]. The condition may be different for single stance phase.

As expected, step width (L) and (R) decreased and increased respectively as turning angle increased. These opposing results were caused by turning: since the walking trajectory and the step length were determined, the foot positions were relatively fixed in the experiment. The subjects had to adjust their step width to complete turning. The maximal absolute value of the ML COM acceleration, ML angle (ankle) and ML angle (FCP) occurred during left (outer) foot stance phase according to Fig. 5. It was believed left foot stance phase was more unstable than right foot stance phase. Therefore, step width (R) corresponding to left foot stance phase should be used to indicate the ML instability of the gait.

Here, as ML stability decreased, step width (R) increased significantly. A larger step width demonstrated a larger deviation of the body from the stance foot. This was assumed to be how the wide steps were correlated to the ML stability in this study.

The maximal absolute ML COM-COP inclination angle was used as an indicator of walking instability [19], because it reflected the degree to which the COM was away from the BOS. It was discovered that the ML MAX (FCP) increased significantly as the curvature of the trajectory increased (Fig. 10). The ML MAX (FCP) was the ML angle when the body was tilting inside most during walking. As the positive direction was inside the circular trajectory, the larger ML angles for a larger curvature of turning meant more inside tilting of the body. This was probably a cause of turning: the body moving towards the inside provided the acceleration required for turning. Another study on curved walking found that the trajectories affected the trunk inclination significantly and the trunk inclined to the inside of the circle during curved walking [28].

A larger ML MAX (FCP) convincingly indicated more difficulty in keeping balance. The highest risk of falling was when the inside leg was swinging (Fig. 5). The results suggest that ML stability increased significantly as the curvature decreased.

A similar finding has been reported by Orendurff et al. that during turning, the ML ground reaction force impulses shifted the body to the inside of the circular trajectory while the impulses during straight walking shifted the body towards the contralateral limb [29]. Additionally, the statistical results of ML COM ACC MAX (Fig. 6) and step width (R) (Fig. 8) coincided with the result of ML angles (FCP) and verified the accuracy of ML angles (FCP) in determining ML stability. It is speculated that the key parameter that affects ML stability is the turning angle in one step. In fact, the change of the step length in circular walking demonstrates the change of turning angles.

In general, the COM- FCP inclination angle and the COM-ankle inclination angle are consistent in the shape and the statistical result: the curves change in a similar manner, the ML MAX (FCP) and the ML MAX (ankle) increase significantly with step length for circular walking or with curvature.

The proposed method accounts for the variability in the BOS, from the heel to flat foot and then the forefoot [30], while the ankle marker is fixed during single stance phase. Additionally, based on Figs. 9 and 10, the proposed method is more sensitive to any change in stability than the COM-ankle inclination angle: more significant differences are found between the ML MAX (FCP) of different tasks.

The COM-ankle inclination angle is only applied during the single stance phase [21]. The double stance phase is more stable than the single stance phase for normal walking, which is the reason why the COM-ankle angle is able to indicate the walking stability. This angle distinguished the elderly fallers from the elderly controls on the condition that the falling only occurred during single stance phase. However, this method may overlook the balance perturbations during double stance phases, for example, during slipping. Therefore, the consideration of double stance phase is one of the advantages of the COM-FCP angle.

The COM-COP angle has been used in some straight walking tasks as the main method [19] or the control methods [21, 31]. The maximal value of the COM-COP angles in the references are around 4° during the single stance phase, and the ML Angle (FCP) in the present study is around 4.5° for normal straight walking (Fig. 10). Only a few researchers have used the COM-COP angle in turning or circular walking tasks [32, 33]. The peak ML COM-COP angle is around 7.0° for healthy subjects, while the ML angle (FCP) in our study for CW1 m with NSL is around 6.9° (Fig. 10). Although the angles are approaching, the tasks are quite different: the subjects completed a 180° turn within the force plate (58 cm × 46 cm) in the reference, where the turning angle in one step should be much larger than that in our study. As deduced in the present result that the ML angle (FCP) increases with the turning angle, it is believed that the ML angle (FCP) should be larger than ML COM-COP angle in the 180° turn, which predicts that the ML angle (FCP) should at least have similar ability to distinguish between different levels of stability as the COM-COP angle.

During the experiment, footprint indicators were used to generate different step lengths. It is inevitable that this may change other gait parameters to different degrees, such as step width and walking direction. The FCP calculation proposed in this study gave a rough estimate of the actual BOS center. Similar to the method using the lateral ankle marker [21], the proposed method leads to a shift in the inclination angles compared to the COM-COP angles. Although the results of these three methods are different, they are all able to indicate ML stability during walking. The sample size was limited to only 10 people, and they were all young healthy subjects. This limited the application of the proposed method. As the changes in stability in young adults completely differ from those in older adults, the application of the present method to elder people and patients should be further investigated. Another limitation of the study was that it failed to account for the learning effect of the central nervous system (CNS). The CNS has the ability to adapt to and learn from new tasks. It is known that humans learn to stabilize unstable dynamics using skilful strategies [34]. Subjects may have learned to stabilize new turns on the basis of the former turns. However, it is believed that any stability improvement requires a number of trials. Therefore, it was felt appropriate to discount any learning effect of the CNS in the analysis.

The proposed method could be used to detect the ML stability of people with smaller step lengths for continuous turning, where force plates are not available. It may also inspire new methods for the monitoring of ML stability for risks of falling. The analysis of turning implies that longer steps or sharper turns during circular walking decrease ML walking stability significantly. These findings may drive the adoption of a series of compensatory strategies aimed at increasing ML stability associated with turning, such as shortening the step length and decreasing the turning angle.

## Conclusions

In this study, a new method to calculate the COM-FCP inclination angle was proposed and verified by ML COM acceleration, step width and COM-ankle inclination angle. The COM- FCP inclination angle was compared among different walking tasks for healthy subjects. The ML COM-FCP inclination angles indicate that ML walking stability decreases with increasing curvature of walking trajectory or step length for circular walking. However, there is no significant difference caused by step length during straight walking. It is deduced that walking stability is affected by the turning angle in one step, not curvature or step length alone.

The proposed method does not require force plate input, is more suitable for people with smaller step length and accounts for variability of the BOS and varying stability during double stance phase. The exploration of inclination angles in different tasks will help further understand ML stability for circular walking and prevent falls in clinical use.

## Abbreviations

BOS: base of support
COM: center of mass
COP: center of pressure
CW1m: circular walking with radius of 1 m
CW2m: circular walking with radius of 2 m
FCP: foot contact point
LSL: larger step length
ML: mediolateral
ML COM ACC MAX: maximal mediolateral acceleration of center of mass
ML MAX: maximal values of mediolateral angles
NSL: normal step length
SSL: smaller step length
SW: straight walking
